# Web alert: Microbial biomass conversion

**DOI:** 10.1111/1751-7915.14149

**Published:** 2022-09-28

**Authors:** Lawrence P. Wackett

**Affiliations:** ^1^ Department of Biochemistry, Molecular Biology & Biophysics BioTechnology Institute University of Minnesota St. Paul Minnesota USA

## Biomass to hydrogen

### https://www.energy.gov/eere/fuelcells/hydrogen‐production‐microbial‐biomass‐conversion

This U.S. Department of Energy site focuses on the use of microbial fuel cells to convert biomass to hydrogen as a source of chemical energy.

## Microbial fuel cells

### https://bioresourcesbioprocessing.springeropen.com/articles/10.1186/s40643‐021‐00365‐7

This is a review article on converting biomass using microbial fuel cells.

## Lignocellulose to platform chemicals

### https://www.sciencedaily.com/releases/2020/08/200827141331.htm

This page describes a process whereby cellulose is broken down by fungi and the sugars converted to lactic acid. Lactic acid, in turn, serves as the microbial feedstock for the production of other commodity chemicals.

## Best chemicals to make using biomass

### https://acee.princeton.edu/acee‐news/study‐ranks‐best‐chemicals‐to‐make‐using‐biomass/

A critical issue in biorefining is to determine the best targets chemicals to make microbiologically. One criteria examined is the savings in greenhouse gas emissions by making a given chemical biologically in comparison with a fossil‐fuel derivation.

## Global biorefinery 2022

### https://task42.ieabioenergy.com/publications/global‐biorefinery‐status‐report‐2022/

This site provides a comprehensive resource regarding biorefineries: How many exist, the types, which feedstocks they use, and what products are generated.

## Bioindustrial manufacturing

### https://www.biomade.org

BioMADE is a relatively new centre with a mission to promote U.S. bioindustrial manufacturing at a competitive scale. Most projects are based on microbial biotechnology research which is then scaled for practical use.

## Biomass distribution on earth

### https://www.pnas.org/doi/10.1073/pnas.1711842115

This broad‐based review article examines the biomass composition of earth with respect to taxonomy, geographic location and trophic levels.

## Catabolism of lignin aromatics

### https://www.pnas.org/doi/10.1073/pnas.1921073117

The ability to use lignin as a source of carbon in manufacturing would be highly significant. The present study used *Pseudomonas putida* as a platform for synthetic biology, building on natural abilities to metabolize lignin aromatics.

## Lignin‐based industries

### https://www.sciencedirect.com/science/article/pii/S1096717621001828

This review article comprehensively examines developments in the microbiological conversion of lignin, which it describes as ‘the world's most underutilized renewable’.

## Cellulosic biomass to fuels

### https://pubs.rsc.org/en/content/articlehtml/2022/ee/d1ee02540f

It is considered here that both microbiological and hybrid biological/catalytic processes for lignocellulose conversion may ultimately prove to be economical.

## Processive cellulase

### https://pubs.acs.org/doi/10.1021/acscatal.1c03465

Cellulase reacts processively along a cellulose polymer and also acts as part of a multi‐enzyme complex to efficiently deconstruct the biopolymer.

